# 17β-estradiol biosensors based on different bioreceptors and their applications

**DOI:** 10.3389/fbioe.2024.1347625

**Published:** 2024-01-31

**Authors:** Xinyi Wang, Fanli Kong, Yaoyao Liu, Shiya Lv, Kui Zhang, Shutong Sun, Juntao Liu, Mixia Wang, Xinxia Cai, Hongyan Jin, Shi Yan, Jinping Luo

**Affiliations:** ^1^ State Key Laboratory of Transducer Technology, Aerospace Information Research Institute, Chinese Academy of Sciences, Beijing, China; ^2^ School of Electronic, Electrical and Communication Engineering, University of Chinese Academy of Sciences, Beijing, China; ^3^ Obstetrics and Gynecology Department, Peking University First Hospital, Beijing, China; ^4^ Department of Thoracic Surgery II, Peking University Cancer Hospital & Institute, Beijing, China

**Keywords:** 17β-estradiol detection, immunosensor, aptamer biosensor, nanomaterials, electrochemistry

## Abstract

17β-Estradiol (E2) is a critical sex steroid hormone, which has significant effects on the endocrine systems of both humans and animals. E2 is also believed to play neurotrophic and neuroprotective roles in the brain. Biosensors present a powerful tool to detect E2 because of their small, efficient, and flexible design. Furthermore, Biosensors can quickly and accurately obtain detection results with only a small sampling amount, which greatly meets the detection of the environment, food safety, medicine safety, and human body. This review focuses on previous studies of biosensors for detecting E2 and divides them into non-biometric sensors, enzyme biosensors, antibody biosensors, and aptamer biosensors according to different bioreceptors. The advantages, disadvantages, and design points of various bioreceptors for E2 detection are analyzed and summarized. Additionally, applications of different bioreceptors of E2 detection are presented and highlight the field of environmental monitoring, food and medicine safety, and disease detection in recent years. Finally, the development of E2 detection by biosensor is prospected.

## Graphical Abstract

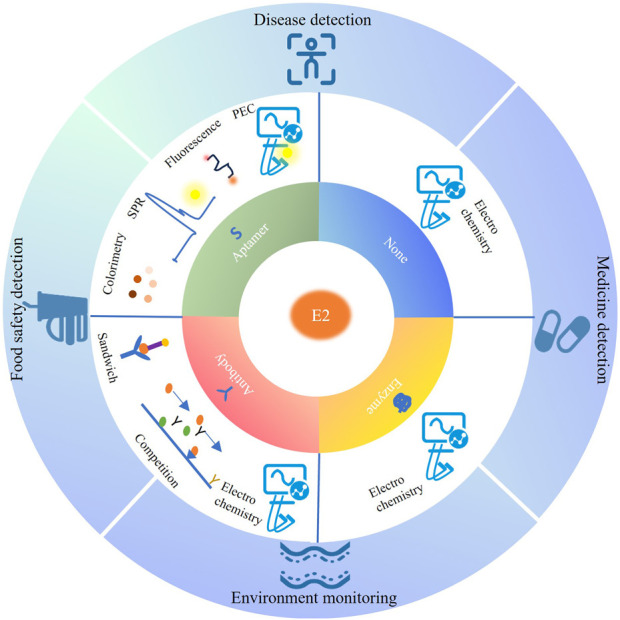

## 1 Introduction

17β-Estradiol (E2) is a critical sex steroid hormone secreted by the mammalian ovary (Frick et al., 2015), primarily in endometrium cells and follicular granulosa cells. Furthermore, considerable evidence supports that E2 is a neuromodulator of learning and memory in human brain (Taxier et al., 2020). As one of the potential environmental endogenous estrogens, human and mammal urine and feces contain E2 (Zhang et al., 2014; Tang et al., 2022b; Tang et al., 2022a). Due to human and animal excretions, E2 can contaminate water resources and aquatic animals through the sewage treatment plant outlet or man-made direct discharge (Liu et al., 2012a). In addition, on farms, the application of animal excretions as fertilizer disrupts the environmental balance of the soil (Liu et al., 2012b). These situations allow E2 to enter the food chain, posing a risk of ingestion by humans (Supchocksoonthorn et al., 2021). E2 has been shown to have significant effects on the endocrine systems of both humans and animals (Dubey and Jackson, 2001). Even at deficient concentrations, E2 can be harmful to humans. For example, the abnormal level of E2 in the human body can damage the endocrine system, cause adverse effects on the development and maintenance of the female reproductive system, obstruct the realization of essential physiological functions of some organs, growth, and endangering the health of future generations (Qiaoxuan et al., 2016). Therefore, detecting E2 sensitively in biological samples is essential for monitoring human, animal, and ecological health.

Currently, the primary methods to detect E2 include liquid chromatography-mass spectrometry, high-performance liquid chromatography, gas chromatography-mass spectrometry. These methods are able to accurately detect E2 in samples with a low limit of detection (LOD) and high specificity. However, most of these methods require complex pretreatments and high-skilled staff, and some produce environmentally harmful organic solvent waste (Nameghi et al., 2019). To meet the requirements of E2 detection in daily life, sensor equipment must be fast, efficient, portable, and easy to operate. In the last few decades, owing to the development of biosensing technology, biosensors can meet all the mentioned requirements, gradually replacing conventional analytical techniques for E2 detection.

The biosensor converts target recognition into a quantifiable and processable signal through the biological reaction between the target analyte and the identified component (Lim et al., 2010; Liu L. S. et al., 2021). The biosensor consists of two functional components: a bioreceptor and a transducer (Sassolas et al., 2012; Crivianu-Gaita and Thompson, 2016). In the first part, using a bioreceptor is one of the methods used in nature to solve the problem of molecular sensing in complex environments (Gerstein and Krebs, 1998; Vallée-Bélisle and Plaxco, 2010). The ideal bioreceptor should not only be able to recognize effectively and bind specifically to the target analyte but also achieve fast response and stable performance, meeting ultra-high selectivity, stability, low detection limits, and other parameters of the biosensor (Zhang et al., 2000). In the second part, the physical transducer converts the splitter binding events into physical signals. A number of new materials are applied to combine transducers, including carbon materials (Metters et al., 2011; Ricci et al., 2012; Pemberton et al., 2013), nanoparticles (Ming et al., 2022), and quantum dots (Steen Redeker et al., 2013; Patterson et al., 2014; Zheng et al., 2015), thus achieving high sensitivity (Liébana and Drago, 2016), high signal conversion efficiency qualitative, and quantitative analysis of biomolecules (Bao et al., 2021; Romero-Reyes and Heemstra, 2021). Hence, the biosensor presents a powerful tool to detect E2 because of its small, efficient, and flexible design.

Environmental endogenous E2 possesses significant physiological roles encompassing metabolic regulation, developmental processes, neural regulation, and reproductive functions. Exogenous E2 may jeopardize the ecosystem stability and risks to human health. Biosensors enable the detection of E2 content in various samples in the environment, and allow for sensitive and selective recognitions at the specific tissue level in human body (Naqvi et al., 2023). In the following review, we provided an overview of the breakthroughs in biosensors for detecting E2 from the past few years, and discussed how the fundamental principles of biosensor systems can be adapted to the design of E2 biosensors. In particular, we categorized E2 biosensors into non-bioreceptor biosensors, enzyme biosensors, antibody biosensors, and aptamer biosensors according to different bioreceptors. We introduced the various forms of transductions corresponding to E2 biosensors based on different bioreceptors and gave specific application examples. Furthermore, we highlighted key advantages, disadvantages, and design points of different bioreceptors for E2 detection. Additionally, a few applications of different bioreceptors of E2 detection were presented and related to the field of disease and environmental monitoring, food and medicine safety in recent years. Finally, we highlighted existing bottlenecks and presented our perspective on the further development of E2 biosensors. The overall framework of this review is illustrated in the Figure-Graphical Abstract.

## 2 Various biosensors for detecting E2

Biosensors offer distinct advantages in rapid and reliable analysis of target analyte. A critical step in designing and optimizing biosensors for detecting E2 is the selection of a bioreceptor. A proper bioreceptor can help a biosensor achieve a fast response and stable performance. The most prominent bioreceptors include enzymes, all monoclonal antibodies (Mabs) (Crivianu-Gaita and Thompson, 2016), aptamers, peptides, and carbohydrate-binding proteins. What should be considered when selecting a bioreceptor for E2 detecting are (1) the affinity with E2; (2) the fixation mode and concentration on the transducer surface; (3) the coupling mode between tag molecules and bioreceptors. The selected bioreceptor needs to have a strong affinity with E2 to ensure high specificity in a complex assay environment. Moreover, the recognition molecules bound to E2 should not easily disengage from the transducer surface. The methods of fixing bioreceptors on the surface of the transducer are generally divided into four types: adsorption, covalent bonding, entrapment, cross linking. The choice of these methods depends on the transducer surface properties and the target application. The immobilization method chosen should maintain the biometric and biocatalytic properties of the bioreceptors. Binding the label molecules of unique materials with bioreceptors promotes specific binding, increases signal conversion, and enhances the detection properties of sensors. The coupling ways between tag molecules and bioreceptors are mainly divided into chemical and physical binding. This part aimed at classifying E2 biosensors based on different bioreceptors, including non-bioreceptor biosensors, enzyme biosensors, antibody biosensors, and aptamer biosensors. Typical examples were listed. Additionally, the advantages and disadvantages of these different E2 biosensors were summarized respectively.

### 2.1 Non-bioreceptor E2 biosensors based on electrochemistry

**FIGURE 1:**
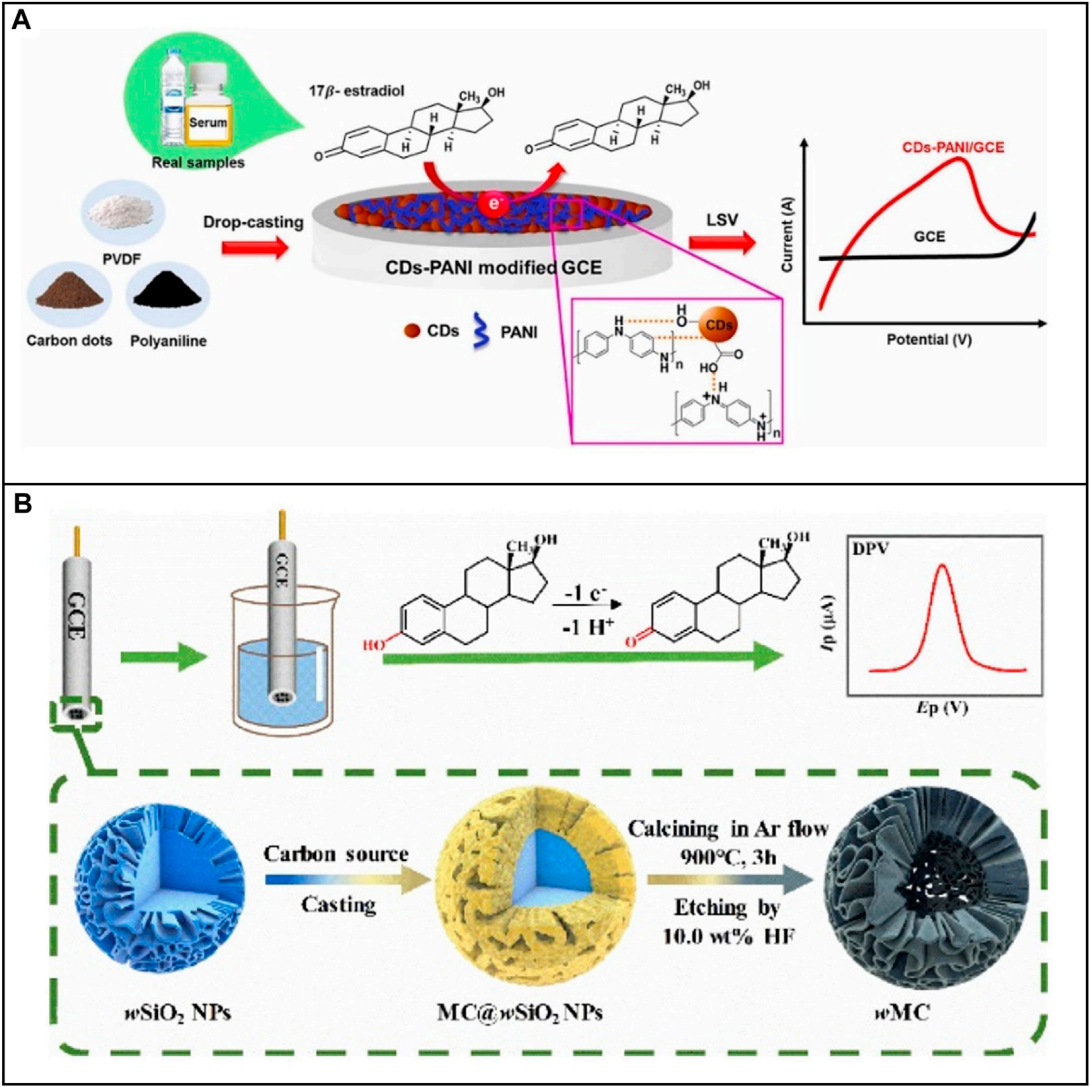
Application of nanomaterials to electrochemical E2 sensors based on non-bioreceptor. **(A)** Electrode modified with nanocomposite CDs-PANI. Reprinted with permission from Supchocksoonthorn et al. (2021). **(B)** Electrode modified with 3D nanocages. Reprinted with permission from Xie et al. (2022).

An advantage of using an electrochemical method to detect E2 is that is its ability to directly sense the electron transfer generated during the oxidation of the hydroxyl group on the E2 molecular structure to the carbonyl group. Hence, this system does not require the design of a bioreceptor. To improve signal quality, a large number of nanomaterials are employed to increase electron transfer efficiency. For example, Nunes Da Silva et al. (2021) designed an electrochemical sensor to detect E2 in milk. This biosensor was based on an MCPE-MMIP (Magneto Carbon Paste Electrode, MCPE; Magnetic Molecularly Imprinted Polymer, MMIP), facilitating pre-concentration, separation, and manipulation of the analyte. This biosensor exhibited sensitivity to E2 across a range from 0.06 to 175 μM with an LOD of 0.02 or 0.06 μM. In another work, Supchocksoonthorn et al. (2021) selected a nanocomposite synthesized by polyaniline (PANI) and carbon dots (CDs) to improve the sensing performance of glassy carbon electrode (GCE) on detecting E2 (Figure 1A). The surface chemical properties of CDs are stable, and there are various functional groups for E2 to attach. Conductive polymer PANI increased the conductivity and sensitivity of this electrode. Cyclic voltammetry (CV) and linear sweep voltammetry (LSV) were used to record the electrochemical detection of E2 utilizing CDs-PANI/GCE across the range of 0.001–100 M with an LOD of 43 nM. More examples of application of nanomaterials in E2 electrochemical sensors are listed in Table 1.

**TABLE 1:** A list of application of nanomaterials to E2 electrochemical sensors without bioreceptor.

Electrode	Modified material	Detection method	Detection range (nM)	LOD (nM)	Sample	Reference
MCPE	MMIP	DPAdsv	60–1.75 × 10^5^	60.00	Water; Milk	Nunes Da Silva et al. (2021)
GCE	25CDs-PANI	CV; LSV	1–1 × 10^5^	43.00	Serum; Water	Supchocksoonthorn et al. (2021)
SPEs	CuPc-P6LC-Nafion	DPV	80–7,300	5.00	Water; Human urine	Wong et al. (2019)
GCE	GQDs/PSSA/GO	DPV	1–6,000	0.23	Serum; Medicinal preparation	Arvand and Hemmati (2017)
GCE	wSiO_2_NP-wMC	DPV	50–8 × 10^4^	8.30	Water; Milk	Xie et al. (2022)
CPE	CeO_2_NPs	SWV	10–1,200	4.30	Water	Souza et al. (2022)
SPE	Fe_3_O_4_/LD-MMIPs	HPLC	3.67–734.27	1.028	Water	Wang et al. (2022)

^a^ Differential Pulse Adsorptive Stripping Voltammetry, DPAdsv; Carbon Dots, CDs; Screen-printed Electrodes, SPEs; Copper Phthalocyanine, CuPc; Printex 6L carbon, P6LC; differential pulse voltammetry, DPV; graphene oxide, GO; graphene quantum dots, GQDs; Carbon Paste Electrodes, CPE; cerium oxide nanoparticles, CeO_2_ NPs; Square Wave Voltammetry, SWV; Daisy-like Magnetic Molecularly Imprinted Polymers, LD-MMIPs; Liquid Chromatography, HPLC.

In addition to the flexible utilization of nanoparticles, electrodes can also be modified structurally to increase the performance of electrochemical biosensing. Nanomaterials have a higher surface-to-volume ratio. Moreover, their three-dimensional networks assembled by one-dimensional or two-dimensional nanostructures can present porous structures (Li et al., 2011). This property maintains a larger area of the active surface, enabling the electrode to attach more biomolecules to a confined active region. For example, Xie et al. (2022) established GCE for the assay of E2 based on the change of the surface nanostructure (Figure 1B). The team used wrinkled mesoporous carbon (wMC) nanomaterials to provide abundant active electron and proton transfer sites. They selected wrinkled silicon nanoparticle (wSiO_2_NPs) as supporting and sacrificial hard templates to form wMC nanomaterials with core-shell nanocage structures, thereby increasing the surface area, electron diffusion, and the number of attachment points for E2. More examples of nanostructure application in E2 electrochemical biosensors are listed in Table 1.

The electrochemical sensor based on non-bioreceptor mostly constructs a primary electrochemical detection platform in E2 detection without incorporating specific bioreceptors. Additionally, the electrode can be modified with specific nanomaterials based on diverse detection requirements. However, because of the absence of biometric components, the electrochemical sensor based on non-biometric molecules performs poor selectivity. If substances with redox molecular structure similar to E2 or substances with redox peak that overlap with E2 are present, the biosensor may be falsely identified as E2, thereby affecting the detection results.

### 2.2 Enzyme E2 biosensors based on electrochemical electrode

E2 biosensors that utilize enzymes as bioreceptors are often combined with electrochemistry. The performance of such biosensors depends on the properties and architecture of the employed enzymes and the surface characteristics and structure of the electrodes (Spychalska et al., 2020). In addition, the way to immobilize the enzyme affects the sensing performance. The immobilized enzyme not only needs to maintain a tight binding to the electrode surface but also ensure its biological activity. There are two commonly used methods for immobilizing enzymes: directly onto the surface of the transducer or by first immobilizing them with a carrier and then attaching them to the transducer (Nguyen et al., 2019). The main methods mentioned above include adsorption, entrapment, covalent crosslinking, chemical crosslinking, and embedding.

Adsorption is one of the simplest fixation methods, presenting fewer destructive effects on enzyme structure than other methods due to the non-necessity of an additional reagent. However, this method is most susceptible to interference during detection, because it is based on weak bonds such as Van der Waals forces, electrostatic, and hydrophobic interactions (Sassolas et al., 2012). Additionally, some physiological conditions like high temperature or pH, substrate addition may weaken the bond (Sassolas et al., 2012). Thus, it is rarely employed in E2 detection.

**FIGURE 2:**
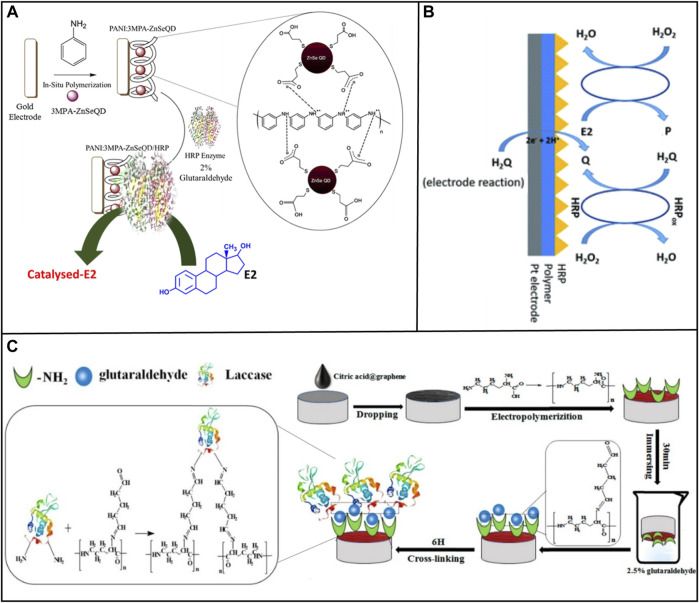
Methods of immobilizing enzyme on electrode surfaces. **(A)** Entrapment. Reprinted with permission from Jijana, (2023). **(B)** Covalent bonding. Reprinted with permission from Spychalska et al. (2020). **(C)** Cross-linking. Reprinted with permission from Wang et al. (2019).

Entrapment requires selecting a suitable polymer to provide the enzyme-free space for dispersion, ensuring high stability, and minimizing leaching (Nguyen et al., 2019). For example, Jijana, (2023) selected Horseradish Peroxidase (HRP) as a bioreceptor to achieve the detection of E2. In this work the nanocomposite 3-mercaptopropionic acid capped zinc selenide quantum dots trapped within the polyaniline (PANI: 3MPA-ZnSeQD) are used to immobilize the HRP on the gold electrode surface by entrapment, as shown in Figure 2A. This biosensor was able to accurately and efficiently measure concentrations of E2 from 0.2 to 4 μM and display an LOD of value 0.2 μM.

Covalent crosslinking presents a powerful method to offer stable complexes between enzymes and supports. Spychalska et al. (2020) used the same enzyme, HRP, as a bioreceptor. The platinum (Pt) electrode was modified by an electroconductive polymer (CP) poly (4,7-bis(5-(3,4-ethylene-dioxythiophene) thiophenes-2-yl) benzothiadiazole), which created an appropriate microenvironment to immobilize the protein and act as a transducer during the transfer of electric charge, as shown in Figure 2B. Then, HRP was immobilized on the surface of the modified electrode by physical adsorption and covalent cross-linking with glutaraldehyde. This biosensor detected the E2 concentration in the medication with a detection range of 0.1–200 M and an LOD of 105 nM.

Chemical crosslinking prevents the active site of an enzyme from being buried in its complex protein structure (Povedano et al., 2017). Wang et al. (2019) modified the GCE with laccase loading with citric acid-graphene (CA-GR) and electropolymerized L-lysine film (Lac/PLLY/CA-GR/GCE), as shown in Figure 2C. Under the best experimental conditions, this biosensor presented a reliable linear detection and LOD. In this system, graphene was treated with citric acid, which solve the problem of the high hydrophobicity and lack of functional group of GR (Liu Y. et al., 2012). Glutaraldehyde was used as a cross-linker to connect laccase and nanocomposite material, generating cross-linking reaction between laccase and nanocomposite material.

Collectively, electrochemical enzyme sensors offer a range of electrode materials, structures, and enzymes to choose from. Various nanomaterials can be incorporated into electrodes to enhance sensing performance. Methods of immobilizing enzymes on electrode surfaces are varied and easy to operate, which are convenient for designing and applying electrochemical enzyme sensors. Furthermore, electrochemical biosensors based on enzymes have the potential to realize the measurement of hormones *in vivo*, which is one of the directions worth developing in the future. However, enzymes are often buried in complex protein structures or modified electrode materials, thus affecting the detection performance. Therefore, it is necessary to select appropriate enzymes and immobilization methods to avoid this situation. Moreover, the electrode surface is structurally modified or modified of nanomaterials with biologically active molecules (Rodriguez-Abetxuko et al., 2020), enhancing the electrochemical reactivity of biomolecules and promoting electron transfer, signal amplification, and enzyme immobilization.

### 2.3 The E2 immunosensor based on multiple detection mechanism

For biosensors, the creation of biosensors *in vitro* and *in vivo* has been dramatically promoted due to the high affinity and specificity of immunoglobulin (Ig) or antibody to its target analyte (Crivianu-Gaita and Thompson, 2015). How the immune sensor performs primarily depends on three key factors: (1) the selection of the antibody; (2) the method of antibody fixation; (3) the biological tag used to label the antibody for signal recognition.

The sensitivity and stability of immunosensors largely depend on the number of surface-fixed immune molecules, their conformational stability, and their orientation on the surface of the sensor (Makaraviciute and Ramanaviciene, 2013). These characteristics are also determined in part by the type of antibody selected. For instance, immunoglobulins are asymmetric molecules whose recognition sites occupy different spatial positions during different fixation processes, resulting in blocked interaction with the analyte (Makaraviciute and Ramanaviciene, 2013). The method of antibody fixation also plays an important role in immunosensors. There are two main approaches that can be used in antibody-based sensor surface preparation: random and site-directed antibody immobilization. Random immobilization mainly includes physical adsorption, covalent immobilization. These methods excel in easy immobilization procedure, but frequently suffer from random orientation of antibody, resulting in high steric-hindrance and poor reproducibility (Kausaite-Minkstimiene et al., 2010). Therefore, building a well oriented layer of antibodies is necessary. Site-directed antibody immobilization technique perform well in this respect, avoiding randomness that occurs when antibody is fixed. The most commonly used technique is to modify coatings on the sensing surface, such as self-assembled monolayers (Zhou, 2006), dextran or various polymers (Kyprianou et al., 2013), to immobilize biomolecules on the sensing surface. To convert the binding events into physical signals the biological tags, such as metal nanoparticles, conductive polymer, fluorescent molecule, are employed. Moreover, these biological tags also can enhance the performance of immunosensors.

According to the design points mentioned above, immunosensors often use a double-biomolecular sandwich mechanism, competition mechanism, and unlabeled electrochemistry to measure E2, which were described in this part.

#### 2.3.1 Double-biomolecular sandwich mechanism

**FIGURE 3:**
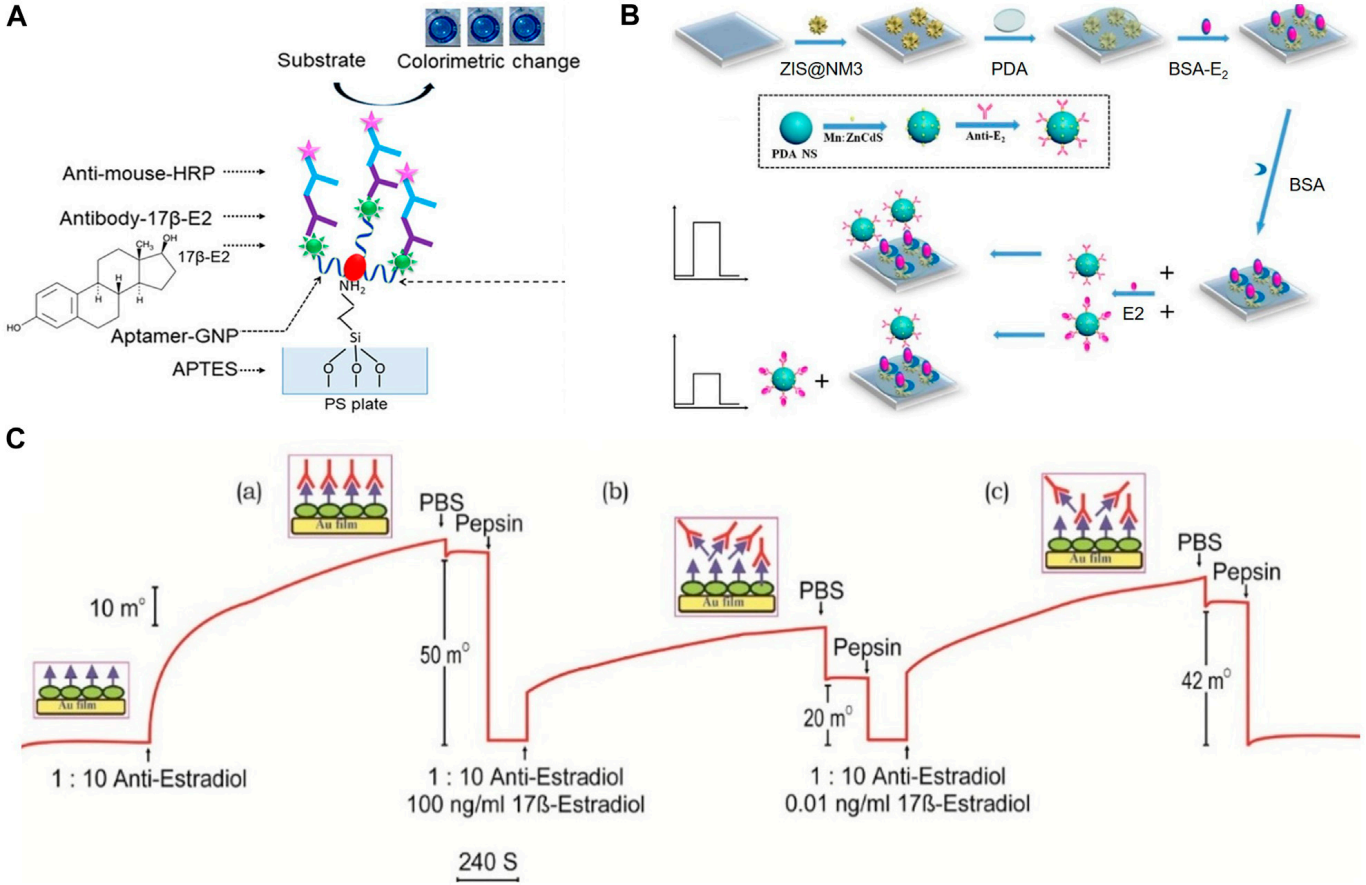
Detection mechanisms for E2 detecting of immunosensors. **(A)** Double-biomolecular sandwich mechanism. Reprinted with permission from Huang et al. (2021). **(B)** Photochemical sensing based on competition mechanism. Reprinted with permission from Yan et al. (2020). **(C)** SPR sensing based on competition mechanism. Reprinted with permission from Kumbhat et al. (2019).

In previous studies, E2 detection using antibody biosensors relied on the binding of human estrogen receptors to estrogen substances in environmental samples, which called enzyme-linked receptor assay (ELRA) with an LOD of 0.1 mg/mL (Seifert et al., 1999). This method typically sandwiches E2 between two biomolecules to detect E2. Further enhancement of sensitivity and signal conversion is achieved by modifying the biological tag on a bioreceptor. For instance, Huang et al. (2021) designed a biosensor for the quantitative detection of E2 by employing an aptamer-E2-E2 antibody sandwich pattern. In this design, an aptamer conjugating with gold nanoparticles captured the probe, while the antibody facilitated detection, as illustrated in Figure 3A.

While E2 biosensors based on the double-biomolecular sandwich mechanism exhibit robust specific recognition ability, a wide range of biometric molecular combinations, and various binding sites for markers. However, the detection process using this mechanism is intricate, and finding two biomolecules capable of binding to both sites of E2 proves to be a challenging aspect.

#### 2.3.2 Competition mechanism

Antibody biosensors based on competitive mechanisms excel in E2 detection. Competitive immunosensors are able to combine with multiple types of signal-sensing units. Xia et al. (2022) constructed a dual-mode electrochemical competitive immunosensor to detect E2. In their study, an electrode served as the signal-sensing unit. The E2 antibody (E2-Ab) was fixed on the composite-modified electrode. The E2-conjugated bovine serum albumin (E2-BSA) was then labeled with a copper-based metal-organic framework (Cu-MOF) and competitively bound with E2-Ab. Saeed Ebrahimi Liu et al. (2007) developed a paper chip sensor based on lateral flow immunochromatography assay (LFIA) to detect E2 in sheep serum using the principle of immunocompetition. First, the purified polyclonal anti-E2 antibody from a New Zealand male white rabbit was conjugated with colloidal gold to form gold nanoparticles (AuNPs) labeled-polyclonal antibody (PcAb). Second, The AuNPs labeled- PcAb was dried and fixed in a conjugate pad at a low temperature to combine with E2. IgG was then covalently immobilized on a nitrocellulose membrane as a control line, used to capture the conjugate of AuNPs labeled-PcAb and E2. Finally, Thyroglobulin (THY) and E2 conjugated by carbodiimide reaction were fixed on the test line to capture AuNPs labeled-PcAb. The test line and the control line formed a competition relationship, and the negative test results generated two red lines (the test and control lines). As the E2 concentration in the sample increased, the test line weakened, providing corresponding results. The LOD of the dipstick was 1 ng/mL. Occasionally, detection results are influenced by factors, including solvent, charged molecules, salt, pH, and temperature. Additionally, auto-aggregation of colloidal nanoparticles probably leads to false-positive or false-negative results (Ghosh and Pal, 2007). However, immobilizing nanoparticles on the paper chip not only mitigates the impact of auto-aggregation (Zhang et al., 2018), but also expands practicality due to simple production and storage.

Furthermore, immunosensors based on competitive mechanisms can apply various forms of signal transformation to complete E2 detection. Yan et al. (2020) integrated the immunocompetition detection mechanism into Photoelectrochemical (PEC) sensing. The electrode was composited with ZnIn_2_S_4_@NH_2_-MIL-125(Ti) (Zinc Indium Sulfide@Amino-functionalized Metal-Organic Framework 125 (Titanium)), as shown in Figure 3B. The linear range was 0.0005–20 ng/mL, and an LOD was 0.3 pg/mL. Kumbhat et al. (2019) developed a Surface Plasmon Resonance (SPR) -based immunosensor using an indirect competitive inhibition immunoassay for E2, as shown in Figure 3C. A competitive relationship existed between E2 and E2-BSA. The detection limit was 1 pg/mL. Additionally, as an immunosensor based on SPR technology, Jia et al. (2018) spin-coated chitosan on the surface of the metal layer to provide abundant amine groups for covalent attachment of antigen (E2-BSA) and improve the biocompatibility of the metal layer. Furthermore, magnetic nanoparticles (MNPs) were introduced as amplification indicators to improve the sensitivity of the SPR sensor. Their designed SPR immunosensor detected E2 with an LOD of 0.814 ng/mL.

Different physical transducers can be chosen for designing E2 biosensors based on competitive mechanisms. Furthermore, several forms of energy exchange interface for antibody attachment can be chosen to achieve signal conversion, including paper chips, electrodes, metal film surfaces, and others. Among them, the paper chip provides a convenient and effective way to realize E2 detection in daily life. A systematic device of the paper chip has been developed to capture the afterglow in the continuous shooting mode of smartphones to obtain detection results (Yao X. et al., 2019). Moreover, modifying the antibody and its attached physical transduction interface with biopolymer materials similar to chitosan provides abundant attachment sites and increases the biocompatibility of the sensor. Modified nanomaterials further enrich the form of signal transformation and are used as amplifying indicators to improve the conversion rate of the signal.

#### 2.3.3 Unlabeled immunoelectrochemical sensing mechanism

Immunosensing based on competition and double-biomolecular sandwich mechanisms need to modify antibodies to provide measurable signals. The electrochemical method without label modification presents a relatively simple and practical advantage. This method detects changes of current or resistance when E2 combines with an antibody. Additionally, electrodes can be modified with various materials to enhance sensor performance. Singh et al. (2017) employed a miniaturized two-electrode system, rather than the conventional three-electrode system, to measure capacitance changes during antigen-antibody interactions. The E2 detection range was 1–200 pg/mL, and an LOD was 1 pg/mL. Wang Y. et al. (2018) further modified SPE with nanocomposite multi-walled carbon nanotubes/thionine/gold nanoparticles (MWCNTs/THI/AuNPs), which improved the electron transfer rate and amplified the signal. This biosensor was based on paper-based microfluidic for the convenient and sensitive detection of E2, achieving an LOD of 17 pg/mL.

In E2 detection, the unlabeled electrochemical immunosensor realizes the advantages of economy, efficiency, and sensitivity, as well as combines with paper-based microfluidic, which achieves real-time diagnosis.

Immunosensors based on mechanisms of double-biomolecular sandwich, immunocompetition, and unlabeled immunoelectrochemical sensing exhibit different detection forms when employed for E2 detection to meet additional requirements. Extensive antibody library screening techniques (Dkhar et al., 2022) and recombinant antibody technology (Sharma et al., 2016) aid in selecting antibodies with high specificity and affinity to detect the target. Proper procedures of antibody immobilization prevent factors that can impair antigen-binding ability, including degradation, random orientation, and steric hindrance of antibodies on the sensor surface (Makaraviciute and Ramanaviciene, 2013; Welch et al., 2017; Sueda, 2022). Moreover, applying different new materials, including carbon nanomaterials, metal nanomaterials, quantum dots, improves the sensing performance in biocompatibility, signal conversion rate, signal amplification, and other aspects. Divine et al. (2021) team has pioneered an innovative protein design method, seamlessly integrating antibodies into regular nanostructures. This approach not only mitigated the uneven distribution of nanoparticles on IgG but also held significant promise for improving signal transduction, thereby enhancing its potential for clinical applications. However, a number of factors caused by antibodies during production limit the application of E2 immunosensors, including high temperature, abnormal pH, limited shelf life, and irreversible denaturation. These factors make E2 immunosensors fail to do well in stability and repeatability.

### 2.4 E2 aptamer biosensor based on multi-signal sensing

An aptamer is a single-stranded deoxyribonucleic acid (DNA) or ribonucleic acid (RNA) fragment obtained through several rounds of screening in the oligonucleotide library by Systematic Evolution of Ligands by Exponential Enrichment (SELEX) (Zheng et al., 2019). Aptamers can bind to target analyte by folding into two-dimensional or three-dimensional structures, including stem ring, hairpin, pseudoknot, bulge loop, and G-quadruplex (Cao et al., 2018; Shaban and Kim, 2021). Additionally, various functional groups are available to modify the aptamer or to immobilize the aptamer to the transducer surface, including -OH, amine, thiol, biotin (Waifalkar et al., 2022). These various functional groups also provide numerous surface immobilization strategies, such as covalent bonding, electrostatic interaction, avidin–biotin interaction, and the self-assembled monolayer (Radi and Abd-Ellatief, 2021). Generally, different transducer surface materials choose different fixation methods, and the details would be expounded upon in this section. Therefore, aptamer biosensors have higher selectivity, affinity, and stability advantages than ordinary antibody and enzyme biosensors.

**FIGURE 4:**
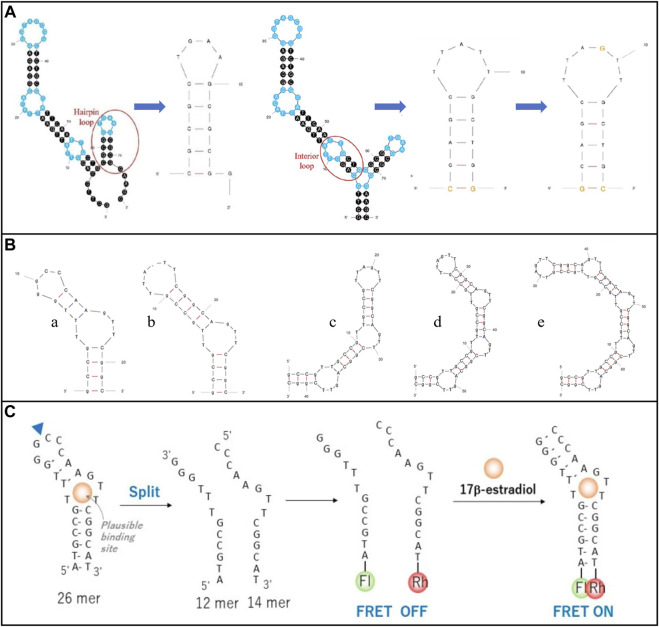
Modification of E2 aptamer structure modification. **(A)** Cut the E2 aptamer redundant bases. Reprinted with permission from Qiao et al. (2021). **(B)** Remove and repeat the E2 aptamer structure. Reprinted with permission from Kongpreecha et al. (2023). **(C)** Split E2 aptamer. Reprinted with permission from Rozi et al. (2022).

The aptamer structure can be modified flexibly to increase the sensing performance (Dong et al., 2014). For example, Qiao et al. (2021) truncated the redundant bases from the E2 aptamer (original E2 aptamer 76-mer (5′-GCT​TCC​AGC​TTA​TTG​AAT​TAC​ACG​CAG​AGG​GTA​GCG​GCT​CTG​CGC​ATT​CAA​TTG​CTG​CGC​GCT​GAA​GCG​CGG​AAG​C - 3′)), as shown in Figure 4A, avoiding the uncertainty in aptamer tertiary structure and instability of affinity for targets in different solutions. This operation effectively increased the compatibility between the aptamer and E2 as well as the performance of the sensor. In Pakawat Kongpreecha team’s work (Kongpreecha et al., 2023), the E2 aptamer was required to wrap gold nanoparticles. Therefore, the original E2 aptamer (original E2 aptamer 22-mer (5′-GCC​GTT​TGG​GCC​CAA​GTT​CGG​C-3′)) structure was removed, and two to five loops were repeatedly added to determine which structure was most effective for E2 recognition. Finally, a specific structure was selected, as shown in Figures 4B, C. In addition, split aptamers are used to strengthen the binding of biometric elements to E2. The aptamer fragment is re-associated in the presence of E2 to restore the desired whole and valid part (Röthlisberger and Hollenstein, 2018). This detection mechanism provides a valuable tool for identifying critical molecular interactions, which avoids effectively generating false positive or non-specific signals (Röthlisberger and Hollenstein, 2018). For example, Rozi et al. (2022) deposited polypyrrole nanowires on carbon screen printing electrode (SPE). They then fixed polymer microspheres on the electrode. Finally, they activated carboxylic acid groups at the end of the microspheres to immobilize E2 split apt12 and apt14, as shown in Figure 4C.

Additionally, aptamers are modified by various tag molecules, including fluorophores, luminescent groups, nanoparticles, and other tags. These modifications link the molecular recognition ability of aptamers with the process of signal generation (Cho et al., 2009; Famulok and Mayer, 2011), thus generating a variety of detectable signals. Then, these detectable signals are easily measured by colorimetry, fluorescence, electrochemistry, and photoelectric chemistry. The above categories were described in this section.

#### 2.4.1 Electrochemical aptamer biosensor

Electrochemical sensing primarily leverages changes in impedance and current for quantitative E2 detection, driven by the conformational changes in the aptamer upon E2 binding or the decomposition and formation of the complex. How electrochemical aptamer biosensors perform depends on the physical and chemical properties of the sensor materials, and selected materials influence the way of immobilizing the aptamer.

Metal electrodes present a powerful tool for detecting E2. For example, Liu M. et al. (2019) used 6-mercapto-1-hexanol (MCH) self-assembled monolayer (SAM) modified Au (MCH/Au) electrode and ferrocene formic acid (FcCOOH) as redox probes to complete E2 detection. This process involved a 60-min incubation of E2 and binding to DNA aptamer. The detection time was 1 min. The complex fixation method causes the incubation time of the sensor to be too long (Waifalkar et al., 2022). Nameghi et al. (2019) recently changed the processing method of metal electrodes and the selection of aptamers. They modified the split aptamer on the screen-printed gold electrode (SPGE) based on the Au-S bond and reduced the incubation time of E2 and aptamer to 30min.

Carbon-based electrodes also demonstrate remarkable performance in E2 biosensing. For instance, Liu X. et al. (2019) utilized gold nanoparticles-thionine-multiwalled carbon nanotube (AuNP-Thi-CNTs) modified GCE, reducing the incubation time to 30 min and achieving a detection range of 12 pM–60 nM with an LOD of 1.5 pM. Moreover, Mat Zaid et al. (2020) successfully decreased the incubation time to 15min by modifying carbon nanodots onto screen-printed carbon electrodes (SPCE). The detection range of E2 was 1.0 × 10^−7^–1.0 × 10^−12^ M, and an LOD was 0.5 × 10^−12^ M.

Various nanomaterials play a crucial role in enhancing electrode modification to improve the sensing performance further. These nanomaterials offer significant advantages, including a large specific surface-volume ratio, size-tunable properties, shape-dependent properties, reduced energy consumption, and a miniaturized size of sensors (Li et al., 2011). These superior characteristics allow nanomaterials to vary their physical and chemical properties through different sizes, compositions, and shapes to meet the needs of different biological interfaces. For example, Ming et al. (2019) designed a folded aptamer sensing platform with microfluidic channels for the electrochemical detection of E2. A novel nanoassemblies of amine-functionalized single-walled carbon nanotube/new methylene blue/gold nanoparticles (NH_2_-SWCNT/NMB/AuNP) was synthesized for the modified electrode with a detection limit of 5 pg/mL. Previous studies using nanomaterials for E2 detection were summarized in Table 2. Electrochemical sensors whose bioreceptor is aptamer performs better by comparing an LOD and detection range. Moreover, electrodes of different materials modified by different nanomaterials advance the performance of the E2 sensor.

**TABLE 2:** A list of E2 electrochemical sensor based on aptamer.

Electrode	Bioreceptor	Method	Detection range (nM)	LOD (nM)	Samples	Reference
25CDs-PANI/GCE	None	CV; LSV	1–1×10^5^	43.00	Serum; Water	Supchocksoonthorn et al. (2021)
CuPc-P6LC-Nafion/SPEs	None	DPV	80–7,300	5.00	Water; Human urine	Wong et al. (2019)
NGQDs/PSSA/GO/GCE	None	CV	1–6,000	0.23	Serum; Medicinal preparation	Arvand and Hemmati (2017)
Ru (dcbpy)_3_^2+^-NCQDs/GCE	Aptamer	CV	1 × 10^-5^-1,000	5.40	Milk powder	Liu et al. (2021b)
PEDOT-GO/Au@Pt/GCE	Aptamer		1 × 10^-4^-1	8 × 10^−5^	Water; Human urine	Zhao et al. (2022)
NH_2_-SWCNT/NMB/AuNP/CPE	Aptamer	CV; DPV	0.0367–1835.6708	0.0184	Serum	Ming et al. (2019)
AuNP-Thi-CNTs/GCE	Aptamer	DPV	0.012–60	1.5 × 10^−3^	Serum	Liu et al. (2019b)
MCH/Au	Aptamer	DPV	7 × 10^-6^-1×10^-2^	5 × 10^−5^	Water	Liu et al. (2019a)
Au	Aptamer	DPV	0.1–7	0.005	Water	Nameghi et al. (2019)
APT-ERGO/GCE	Aptamer	SWV	1 × 10^-6^-9×10^-3^	5 × 10^−7^	Water	Rather et al. (2018)
			1.2 × 10^-2^-0.23			
CDs/SPCE	Aptamer	EIS	1 × 10^-3^-100	5 × 10^−4^	Water	Mat Zaid et al. (2020)

^a^ Nitrogen-doped Carbon Quantum Dots, NCQDs; Poly (3,4-ethylenedioxythiophene), PEDOT; aptamers, APT; Screen-printed carbon electrode, SPCE; electrochemical impedance spectroscopy, EIS.

Aptamer sensors based on electrochemical signal sensing offer a diverse range of electrode structures and materials, providing different forms of signal transformation according to detection requirements, with various detection methods and fast detection speed. Due to the tenacity of the electrode structure and the reversibility of the aptamer’s recognition of E2, the aptamer biosensor based on electrochemical signal sensing is another device used to detect hormones *in vivo*.

#### 2.4.2 Photoelectric chemical aptamer sensor

As a new technology, different from traditional electrochemical sensing, Photoelectrochemistry (PEC) sensing employs light as an excitation source and use electric energy for the readout in PEC sensing, reducing the dependence of potential (Shu and Tang, 2020). The utilization of different energy forms between the excitation source and detection signal allows PEC sensing to exhibit higher sensitivity compared to traditional electrochemical and chemiluminescence methods (Shu and Tang, 2020). Therefore, PEC sensing is widely used in E2 biosensing because of its excellent performance in easy miniaturization and operation, separation of input optical and output electrical signals, low background signals, accurate recognition, and fast response (Feng et al., 2018).

In the process of PEC sensing, the light absorption of photoactive material is the first step to achieving photoelectric conversion and is an essential component of PEC biosensors. Therefore, photoactive material requires excellent light capture capability to meet the detection requirements. People have been working on adjusting photoactive materials to avoid photocorrosion, improving the photogenerated conversion rate, excitation of visible light, and other attributes. This part mainly showed the application of different photoactive materials in the PEC detection of E2.

Photoactive materials such as semiconductors, semiconductor metal oxides, and metal sulfides have gained prominence in recent years due to their excellent chemical durability and photocatalytic activity. However, sometimes only selecting one of these materials probably appears to be a severe photocorrosion phenomenon. According to some researches, combining different materials can solve these problems. For example, Tu et al. (2022) chose cadmium sulfide (CdS) with excellent photocatalytic performance as the photoelectric material. They presented a rose-like nanostructure on the electrode surface to increase the electrode surface area and better bond biomolecules. However, because CdS is prone to aggregation and light radiation, a thin carbon layer was added to solve the problem. The linear range of E2 was 1.0–50 nM, and an LOD was 0.37 nM. This paper used alkaline phosphatase (ALP) to mediate for modifying E2 aptamers to achieve signal amplification.

**FIGURE 5:**
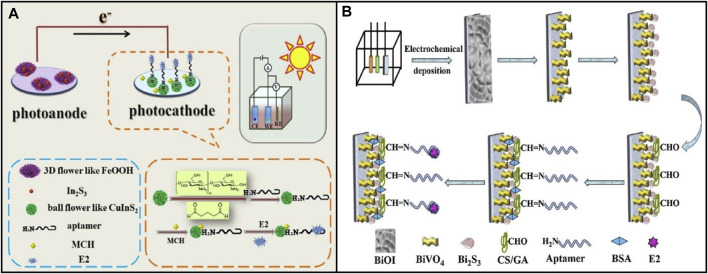
Application of various photoelectric materials in E2 aptamer sensor based on photoelectric chemistry. **(A)** N-type semiconductor FeOOH/In_2_S_3_ and P-type semiconductor CuInS_2_. Reprinted with permission from Li et al. (2020). **(B)** Mo-p BiVO_4_ BiOI nanoarrays. Reprinted with permission from Feng et al. (2020).

Part of the reason for the low photoelectric conversion rate in PEC sensing is electron–hole pairs recombination. Currently, the principles of separating and transferring photogenerated carriers are more useful in the interface of different components. Thus, various strategies have been exploited to inhibit charge-carrier recombination, including designing and constructing semiconductor-semiconductor heterojunctions, semiconductor-carbon heterojunctions, and multi-component heterojunctions (Wang et al., 2014). Li et al. (2020) modified the N-type semiconductor FeOOH/In_2_S_3_ (Iron hydroxide oxide/Indium (III) sulfide) for the photoanode and the P-type semiconductor CuInS_2_ for the photocathode, as shown in Figure 5A. CuInS_2_ is a ternary semiconductor with a high optical absorption coefficient and high stability. The heterojunction between FeOOH and In_2_S_3_ enhanced the transfer of photo-generate electrons to photoanode and promoted the separation rate of electron-hole pairs and the photocurrent response. The LOD was 3.65 fg/mL, and had good selectivity, stability, and reproducibility. Moreover, metal oxides and sulfides are combined, using the heterojunction between them to effectively separate the photogenic charge. For example, Wu et al. (2023) developed a portable biosensor based on PEC, which could visualize real-time monitoring E2. The probe of this biosensor was modified with BiOBr/Ag_2_S. The LOD was 0.18 pM.

In addition, nanotechnology is used to solve the uneven distribution and loose combination of sensor substrate materials, increasing the sensitive surface. Feng et al. (2020) synthesized Mo-doped porous BiVO_4_ (Mo-p BiVO_4_) through chemical and thermal treatment and transformed it into Mo-p BiVO_4_ BiOI nanoarrays by electrodeposition, as shown in Figure 5B. Through a sacrificial synthesis method, Bi_2_S_3_ (bismuth sulfide) nanoparticles were grown *in situ* on the surface of Mo-p BiVO_4_ to form a new Mo-p BiVO_4_/Bi_2_S_3_ heterojunction to obtain a higher PEC signal. In the range of 1 × 10^-3^–5×10^2^ pM, the photocurrent response to target E2 was excellent, and the detection limit was 3.2 × 10^−4^ pM. The nanoarray structure accelerates electron transfer due to a large specific surface area. Moreover, it provides various binding sites to fix biomolecules, thus enabling the biosensor to achieve high sensitivity.

Although the development history of aptamer sensors based on photoelectric chemical signal sensing is relatively short, many researchers have found that PEC biosensors show the advantages of low cost, high sensitivity, and easy miniaturization, which are primarily close to the needs of E2 detection in daily life. PEC sensing evolved from the electrochemical analysis. Compared with electrochemical sensing, photoelectric conversion and corrosion rates of photoactive materials under light are considered when detecting E2. Additionally, the photoactive materials are easily peeled off and polluted in the multi-step modification and detection process. However, it can be improved by the combination of different materials and nanotechnology (Shu and Tang, 2020). Hence, to solve these problems, it is critical to develop new PEC technology to increase the sensing stability so that the aptamer E2 sensor based on photoelectric chemical signal sensing can be removed from the laboratory to the commercial market.

#### 2.4.3 Optical aptamer biosensor

Optical biosensors own significant advantages in the direct and real-time detection of target analysts. The measurement uses different tag molecules to generate optical signals, including colorimetry, fluorescence, raman spectroscopy, and others. This part focused on describing the application of various label molecules in colorimetry, fluorescence, and raman spectroscopy.

##### 2.4.3.1 Colorimetry

In recent years, colorimetry has gained widespread use in the detection of E2 due to its simplicity, rapid response, and high-throughput analysis capabilities. To attain colorimetric functionality and specific selectivity in colorimetric sensors, functional nanomaterials or optical probes are typically required (Yu et al., 2020; Wang et al., 2021). Among these, metal nanoparticles are the most commonly employed. Because of the nano-size effect, different sizes show different colors, widely used in colorimetric sensors. Qiao et al. (2021) employed colorimetric methods to design a biosensor for detecting E2 in serum, urine, or water. The detection mechanism utilized AuNPs to mitigate salt-induced aggregation in a NaCl solution, resulting in the color change of AuNPs to blue or purple. The E2 aptamer was adsorbed on the AuNPs surface by van der Waals forces and DNA base-gold interaction to protect AuNPs from salt-induced aggregation, resulting in the wine-red color of AuNPs. After adding E2, AuNPs was removed from the protection of the aptamer due to the increased affinity between E2 and E2 aptamer. With the increase of E2 concentration, the color of the solution changed from the original wine red to purple or blue. The LOD was 0.02 μg/mL.

The aptamer sensor, based on color change, achieves a simple method and provides intuitive results when detecting E2. Nevertheless, the detection accuracy of colorimetry is inferior to that of other methods, which limits its application range.

##### 2.4.3.2 Fluorescence

Biosensors based on fluorophores present a powerful tool to detect various targets in clinical diagnosis. Fluorescent biosensors consist of two key components: target recognition and signal transduction modules. In signal transduction, nanoparticles are commonly employed as transduction materials to converts the physical and chemical changes generated in the process of target recognition into detectable fluorescence signals. This section focused on the application of various nanoparticles on fluorescent aptamer biosensors for E2 detection.

**FIGURE 6:**
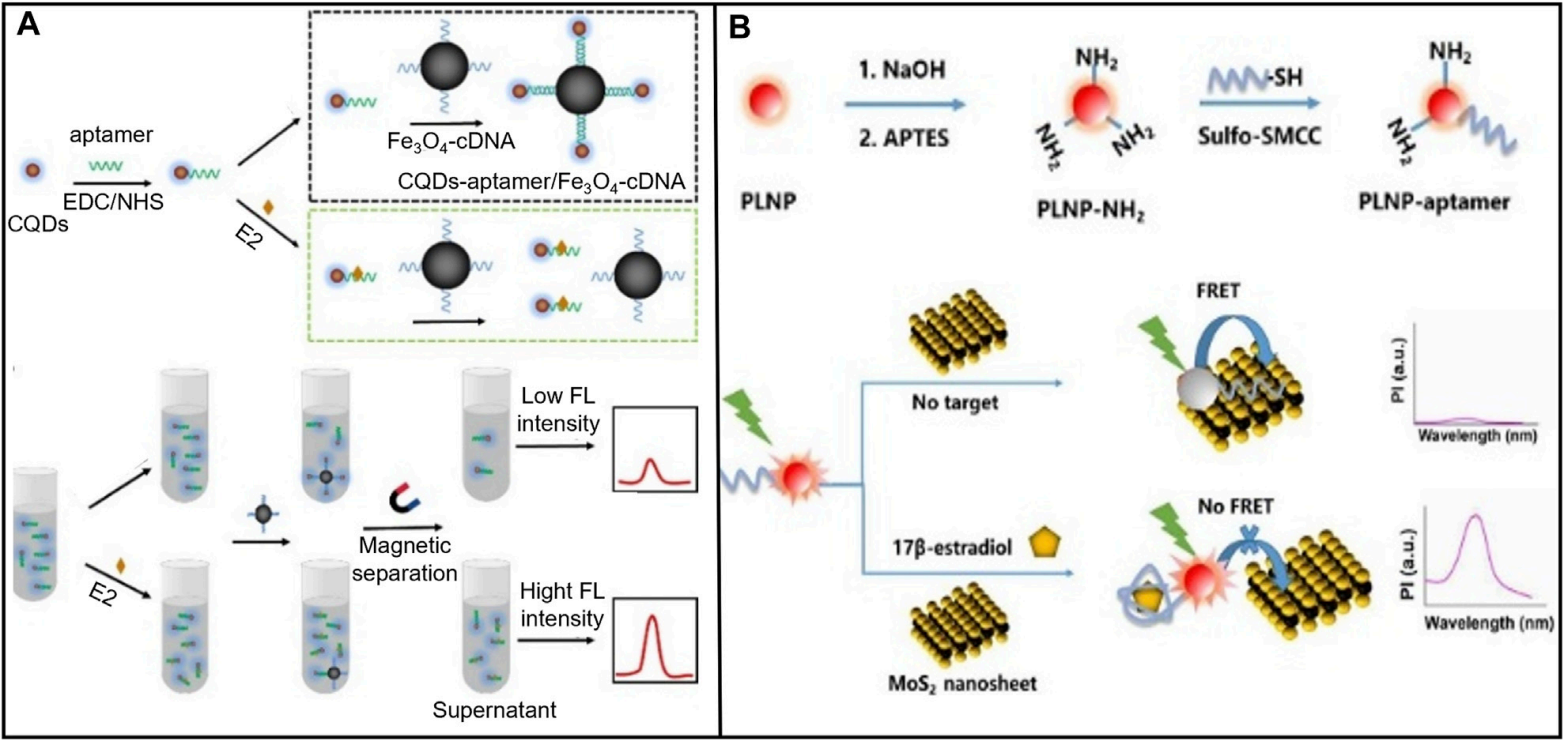
E2 aptamer sensors based on various fluorescent markers. **(A)** CQD was used as a fluorescent marker to modify the E2 aptamer, and the magnetic material Fe_3_O_4_ modified the DNA single strand complementary to the E aptamer. Reprinted with permission from Wei et al. (2022). **(B)** A MOF Ru (bpy)_3_^2+^ molecule was constructed to construct a FRET-based fluorescence measurement sensor platform. Reprinted with permission from Sha and Yan, (2021).

When selecting the fluorescent probe, the aptamer sensor should consider both the stability of fluorescence performance and the binding ability with the aptamer. For example, Wei et al. (2022) selected medium carbon quantum dots (CQD) as fluorescent markers to modify the E2 aptamer and magnetic material ferroferric oxide (Fe_3_O_4)_ to modify the single DNA strand, which was complementary to the E2 aptamer. To hybridize it, they then detected E2 sensitively through fluorescence quenching, as shown in Figure 6A. Among them, CQD was a carbon nanomaterial whose surface was rich in various functional groups to effectively modify E2 aptamers and had stable fluorescence performance. Magnetic nanomaterial Fe_3_O_4_ was used to rapidly separate targets from complex sample substrates, effectively reducing interference from other chemicals. E2 detection was successfully performed in milk samples, with a linear range was 1 × 10^−11^-1×10^-6^ M, and an LOD was 3.48 × 10^−12^ M. Furthermore, the performance of fluorescence sensors significantly depends on the selection of fluorescence quenchers. Improper selection of quenchers probably leads to limited absorption wavelength range, low quenching efficiency, and steric hindrance caused by uncontrollable spatial orientation when the quencher combines with biomolecules. Ren et al. (2019) selected upconversion nanoparticles (UCNPs) as the fluorescent marker and black phosphorus nanoparticle combined with DNA tetrahedron (BP-Au@T-cDNA) as the quenching probe to detect of E2. T-cDNA amplified the signal, and 2D nano complexes composed of BP and Au had higher optical absorption and reduced steric hindrance of cDNA. This system measured E2 in water, food, serum, and urine samples.

In addition, when detecting fluorescence, the experimental conditions and noise interfere with the single signal fluorescence detection (Fang et al., 2016; Su et al., 2019; Sha and Yan, 2021), affecting the accuracy of analytical results. Fluorescence quenching based on fluorescence resonance energy transfer (FRET) mechanism has been found to correct for interference from external factors to improve accuracy (Sha and Yan, 2021). For example, Zhang et al. (2022) covalently combined persistent luminescence nanoparticles (PLNPs) with E2 aptamer to form PLNP-aptamer. PLNP-aptamer was used as a FRET pair energy supplier. Molybdenum disulfide (MoS_2_) nanosheets (a 2D materials) were used as a quencher, as shown in Figure 6B. The detection range for E2 was 0.5–1.2 × 10^3^ ng/mL, with an LOD of 0.29 ng/mL. Sha and Yan, (2021) constructed a FRET-based sensor platform for fluorescence measurement using a metal-organic framework (MOF) molecule, Ru (bpy)_3_^2+^, to estimate E2 quantitatively in serum. The LOD was 0.2 nM. MOF presents well on long excited states lifetime, easy modification, and high porosity. These features make MOF be extensively used for constructing fluorescence sensing platforms.

Aptamer sensors based on fluorescence should pay attention to the selection of fluorescent probes. Nanomaterials can be composed of organics, metal, or both and are less than 100 nm in length along at least one dimension (Zhong, 2009). This small size scale leads to large surface areas and unique size-related optical properties. For example, Fluorescent semiconductor quantum dots exhibit the quantum confinement effect because their very small (<10 nm) dimensions (Smith and Nie, 2004). This effect makes them own wide UV-visible absorption spectra, narrow emission bands, and optical properties that can be tuned by size, composition, and shape (Klostranec and Chan, 2006; Zhong, 2009). Metal nanomaterials not only can be used as fluorescent probes but also used to amplify the fluorescence signal. Metallic nanostructures can interact with proximal fluorophores and produce an increased quantum yield with improved photostability, which improve the sensitivity of fluorescence detection to detect molecules at ultra-low concentrations (Jeong et al., 2018). Two-dimensional nanomaterials exhibit excellent adsorption capacity and extensive UV absorption (Han et al., 2020), including Graphene, transition metal dichalcogenides, black phosphorus, MXenes, and others (Lei and Guo, 2022). Although nanomaterials are promising labels in fluorescent sensing, factors such as water solubility, surface functionalization, and chemical purity still affect the performance of nanomaterials in biosensing. Therefore, the synthetic and modification strategies, analytical characterization and purification are still needing continuous improvement.

##### 2.4.3.3 Surface-to-enhanced raman spectroscopy

**FIGURE 7:**
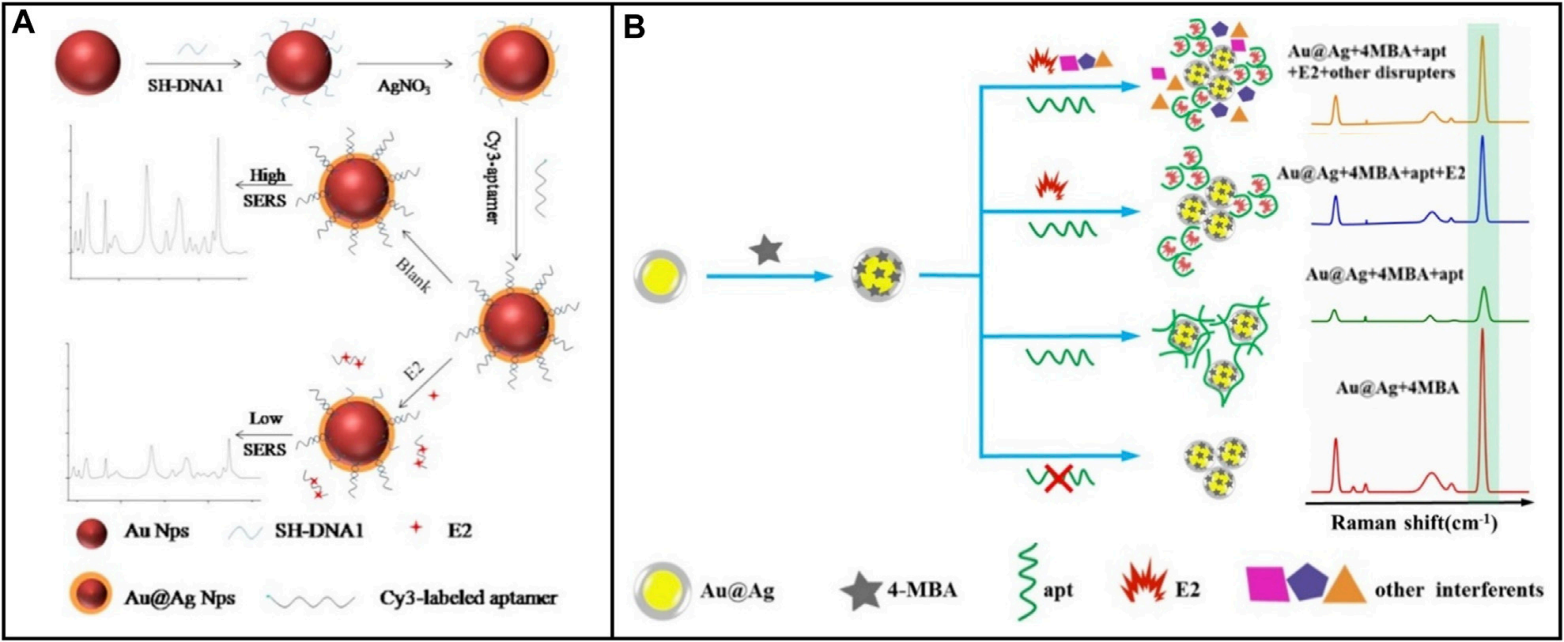
E2 aptamer sensor based on various Raman probe molecules. **(A)** Au@AgCS NPs was used as carrier and 4-MBA was used as Raman probe molecule. Reprinted with permission from Liu et al. (2018). **(B)** Cy3-labeled E2 aptamer and Au@Ag CS NPs were used as Raman probe molecules. Reprinted with permission from Pu et al. (2019b).

Surface-to-enhanced Raman spectroscopy (SERS) has become increasingly popular as a non-damaging detection method compared to other optical methods. SERS-based biosensors use inelastic light scattering to accomplish substance concentration detection. This specific process is that when nanoparticles (NPs) are adsorbed on the corrugated metal surface, this scattering enhances light scattering to detect the target (Kim et al., 2023). SERS presents well in enhancing signal and having narrow peaks, providing high sensitivity and selectivity for the biosensor (Yao L. et al., 2019). Liu et al. (2018) designed a SERS biosensing system that served Au @Ag CS NPs as the carrier, 4-MBA (4-mercaptobenzoic acid) as the raman probe molecules, and E2 aptamer as the recognition element, as shown in Figure 7A. The LOD was 0.05 p.m. However, due to the low raman spectral intensity of small organic molecule E2 (Liu et al., 2018), E2 only is detected at high concentrations. To solve this problem SERS active substrate is improved to generate a high raman signal (Chao et al., 2016), or a substrate labeled with raman probe molecules is used to enhance the sensitivity of E2 detection by SERS. For example, Pu et al. (2019b) developed a SERS biosensor using Au@Ag NPs as the carrier, and E2-aptamer labeled sulfo-cyanine3 (Cy3) as raman probe molecules, as shown in Figure 7B. First, the strand DNA used to attach the E2-aptamer was immobilized to the AuNPs surface by Au-S covalent bonds. Second, AuNPs were coated with AgNPs to enhance raman signaling. Finally, the resulting core-shell nanoparticles Au@Ag NPs were attached with Cy3-labeled E2 aptamers. Cy3-labeled E2 aptamer and Au@Ag CS NPs give SERS high sensitivity and selectivity. The linear range of E2 detection was from 1 × 10^−13^-1×10^-9^ M, and an LOD was 2.75 fM. This work combined AgNPs with a firm surface enhancement effect but poor stability with AuNPs with good stability but relatively weak SERS effect to achieve a win-win effect.

Compared with fluorescence and colorimetric biosensors, the aptamer sensor based on raman spectral signal sensing has a lower background signal in detecting E2, does not appear to be fluorescence bleaching, and achieves multiple repeated measurements. A crucial research focus in SERS-based biosensor development is the recruitment of different nanostructured materials, or their combinations, to enhance the Raman signals generated. Therefore, in the design and application, selecting SERS active substrate and aptamer marker should be fully considered to compensate for the low Raman spectral intensity of E2.

## 3 Multi-field application of E2 biosensor

The pollution caused by environmental endocrine disruptors (EED), endocrine-disrupting chemicals (EDCs), and environmental estrogens (EEs) has become a widespread concern. Estrogen is one of the most common endocrine disruptors. When excessive estrogen enters the food chain, it not only pollutes the environment but also poses risks to public health. E2, which has potent estrogen activity, plays a significant role in the growth of humans and animals. Therefore, it is imperative to use E2 biosensors to quickly and sensitively detect E2 concentration in food, water environment, and the human body. This part primarily introduced the application of various E2 biosensors in aquatic environments, food safety, and disease detection in recent years.

### 3.1 Application of E2 biosensor in aquatic environment monitoring

Steroestrogens (17α-acetylenestradiol, 17β-estradiol, and estrone) have been included in the EU Water “watch list” of the EU Water Framework Directive (WFD) (Kase et al., 2018). Even if the content of E2 is not high in the sewage discharged from livestock farms, sewage treatment plants, and food processing plants, it will endanger human health through the accumulation of the food chain. Numerous studies have demonstrated the widespread presence of E2 in samples from sludge, sediment, rivers, and lakes (Wang et al., 2011). Therefore, timely detecting of water resources, food, and other substances that are directly ingested by the human body ensures the E2 content at a safe level. Currently, biosensors based on enzymes, antibodies, and aptamers have been successfully developed to detect E2 in water resources and food. Some methods are summarized in Table 3.

**TABLE 3:** A list of application of E2 biosensor in aquatic environment monitoring and food safety detection.

Bioreceptor	Detection method	Detection range (nM)	LOD (nM)	Sample	Reference
None	EC	100–23000	MIP: 30	Water	Da Silva and Pereira (2022)
			CB: 100		
None	EC	0.01–100	1.86 × 10^−3^ or 6.19 × 10^−3^	Water	Regasa and Nyokong (2022)
None	EC	1–6,000	0.23	Medicinal preparation	Arvand and Hemmati (2017)
Enzyme	EC	100–2×10^5^	10^5^	Medicinal preparation	Spychalska et al. (2020)
Enzyme	MIP-PM	None	0.9178	Milk; Human urine	Xiao et al. (2017)
Enzyme	Cantilever nanobiosensor	None	0.0015	Water	De Cezaro et al. (2020)
Antibody	ICC	None	18.357	Milk	Wang et al. (2018b)
Antibody	LFICA	1.028–29.371	1.836	Food	Yao et al. (2021)
Antibody	LSPR	0.011–100	0.011	Water	Minopoli et al. (2020)
Antibody	Colorimetry	None	0.7343	Food	Yao et al. (2019b)
Aptamer	Reflectance spectroscopy	3.3 × 10^-3^-0.734	3.3 × 10^−3^	Milk	Jiang et al. (2018)
Aptamer	Fluorescence analysis	0.367–367	0.3378	Water; Food	Ren et al. (2019)
Aptamer	RS	1 × 10^-4^-1	2.75 × 10^−6^	Water	Pu et al. (2019b)
Aptamer	ECL	1 × 10^-5^-1,000	5.4	Milk powder	Liu et al. (2021b)
Aptamer	GFET	5–5,000	0.0347	Water	Li et al. (2019)

^a^ Electrochemistry, EC; Graphene field-effect transistor, GFET; electrochemiluminescence, ECL; immunocompetitive chromatograph, ICC; lateral flow immune competition assay, LFICA; Molecularly imprinted polymer grafted paper-based method, MIP-PM; raman spectroscopy, RS.

It is challenging to detect trace hormones in the environment. Single-use analysis methods with suboptimal LOD may sometimes fail to meet the detection requirements. Optimizing the signal processing circuit, biometric identification, and detection devices can enhance the signal quality. It can be known from the second part of this paper that selecting appropriate bioreceptors and modifying appropriate functional materials effectively increase the sensing performance of the E2 biosensor. Furthermore, various innovative detection devices have been designed to optimize signal quality. The work of Li et al. (2019) was ingenious. They proposed a differential graphene field-effect transistor (GFET) sensor to selectively detect E2 in the presence of nonspecific interference. Certain biosensors are susceptible to nonspecific interference from factors such as pH, ionic strength, temperature, and other parameters. Therefore, Yijun et al. designed two sensing units to address this issue. The measuring unit was sensitive to analytes and interference, and the reference unit was only sensitive to interference. This design minimized the impact of changes in environmental conditions on measurement accuracy, achieving an LOD of 34.70 pM. These results were consistent with the results of non-interference detection. De Cezaro et al. (2020) first developed a cantilever nanobiosensor modified with tyrosinase to detect E2, with a sensitivity of 0.101 V/ug and an LOD of 0.4 ng/L. This nanobiosensor significantly increased the ability to recognize nanoscale events.

### 3.2 Application of E2 biosensor in food and medicine safety

As a natural estrogen, E2 plays an important role in EDCs, exhibiting the strongest estrogenic effect when introduced into the human body exogenously, despite its essentiality to human physiology. When consuming food or medicine with E2 residues or contamination, human beings are exposed to exogenous E2 unconsciously (Pu et al., 2019a). Therefore, it is of great importance to develop rapid, sensitive and selective biosensors for detecting E2 residues in food matrices. Some methods are summarized in Table 3.

Due to the majority of E2 exposure from foods come from animal-derived food, most studies focused on dairy and meat products (Hartmann et al., 1998; Malekinejad et al., 2006; Courant et al., 2007; Regal et al., 2012). Some researches found that positive associations were observed between dairy product consumption and total and free estradiol concentrations (P for trend = 0.02 and 0.03, respectively) (Brinkman et al., 2010). Therefore, although the concentration of E2 in commercial dairy products is usually lower than 100 ng/L, the low dose effect and biological amplification effect of E2 cannot be ignored (Pape-Zambito et al., 2010). The traditional method used to measure E2 in dairy products, such as milk, are hollow fiber-based stirring extraction bar liquid-liquid microextraction (HF-SEBLLME) (Xu et al., 2013), HPLC with magnetic solid phase extraction method (Wang et al., 2015), and so on. Although these methods can accurately detect the content of E2, they need more sample pretreatment and detection times before detection. Xiao et al. (2017) fabricated an activated paper with molecularly imprinted polymers (MIPs) to detect E2. The whole detection process could be finished in 10 min with a much lower cost. More importantly, the result could be directly observed by naked eye. To satisfy E2 concentrations below ng/L level, Jiang et al. (2018) selected aptamer which perform well in thermal stability as bioreceptor, and applied Poly (N-isopropylacrylamide) on the surface of the transducer as a microgel layer to complete the detection of E2 in milk. The detection limit was 0.9 pg/mL (3.2 pM).

Meat products are another food product containing estrogens such as estrone, estradiol, and estriol. Illegal use of hormonal drugs in animal feed to promote growth rate is common, resulting in residual hormones in meat. Therefore, exogenous estrogen may accumulate in the human body through dietary estrogen intake and affect human health. Because meat products are not liquids, the use of portable biosensors also requires a series of pretreatment procedures. At present, HPLC and GC-MS techniques are commonly used to detect the E2 content in meat products.

E2 is also found in many medicines. For example, the concept of using natural E2 in combined oral contraceptives emerged in the 1970s (Greenblatt et al., 1977; Wenzl et al., 1993; Hoffmann et al., 1998). Although the introduction of E2 may reduce cardiovascular risk in women using oral contraceptives, it may also lead to other physical health conditions. In addition, it is also necessary to accurately detect the E2 level during clinical drug intervention. Hence how accurately control the E2 level during drug preparation is important. However, the current detection of E2 in pharmaceutical reagents still largely relies on large-scale equipment like HPLC. There are currently limited reports on the direct utilization of biosensors.

### 3.3 Application of E2 biosensor in disease detection

E2 is a primary female steroid hormone primarily produced in the ovaries, and in small amounts in the liver, heart, muscles, bones, and brain (Cui et al., 2013). E2 primarily regulates physiological functions in various tissues through the estrogen receptor (ER). The growth, development, and differentiation of various cancer cells are accompanied by abnormal E2 content (McDonnell and Norris, 2002; Burns and Stabile, 2014; Che et al., 2014; Huang et al., 2018). E2 protects the cardiovascular system and inhibits the aging of Human Vein Endothelial Cells (HUVECs) (Fang et al., 2018; Xiang et al., 2023). E2 is also believed to play neurotrophic and neuroprotective roles in the brain, including regulating reproductive behavior, spinal density in male and female forebrains (Lu et al., 2019), synaptic plasticity (Brandt et al., 2020), neuroprotection, and hippocampus-dependent cognitive function with memory (Lu et al., 2019).

In conclusion, E2 is extremely important for human health. At present, high sensitivity ELISA kit (Lu et al., 2019) and gas chromatography-mass spectrometry (GC-MS/MS) (Ankarberg-Lindgren et al., 2018) have been used to detect E2 content in human body fluids. However, they require complex preprocessing and highly skilled operations. Therefore, efficient, sensitive, and miniature biosensors are needed to detect E2 content in the human body. The reference range of E2 concentration in urine and serum is essential for clinical evaluation, and part of the detection methods are shown in Table 4. The instruments listed in the table achieved miniaturization, high efficiency, and sensitive detection of E2. Nevertheless, they all need to conduct specific pretreatment of samples, and integration of sampling and detection fails to be formed. The literature reviewed so far is all about *in vitro* detection of E2. Continuous and dynamic *in vivo* examination in clinical application is able to help to monitor the condition and provide better treatment. As previously mentioned in this context, E2 can regulate the plasticity of neural synapses and the formation of memory as a newly discovered neuromodulator in the brain (Lu et al., 2019), thus helping to treat some neurological diseases. However, current research methods are to affect the expression of E2 in the neural circuit by gene knockout or drug intervention (Spencer-Segal et al., 2012; Lu et al., 2019), or to directly remove the gonads to study the effect of E2 on the electrophysiological properties of neurons (Tuscher et al., 2016; Zhao et al., 2018). None of these methods can accurately reflect the concentration of E2 that can produce positive effects on neurological diseases. Therefore, it is also necessary to develop some new biosensors that can detect the changes of E2 concentration in specific brain regions in real time.

**TABLE 4:** A list of application of E2 biosensor in disease detection.

Bioreceptor	Detection method	Detection range (nM)	LOD (nM)	Sample	Reference
Enzyme	EC	4 × 10^-4^-0.057	1.33 × 10^−4^	Human urine	Wang et al. (2019)
Antibody	RS	None	1.8 × 10^−4^	Human urine; sutem	Li et al. (2022)
Antibody	EC	0.0367–36.713	0.0198	Serum	Xia et al. (2022)
		0.00367–36.713	0.00173		
Aptamer	EC	0.01–10	0.002	Human urine	Lin et al. (2012)
Aptamer	EC	1 × 10^-4^-1	8 × 10^−5^	Human urine	Zhao et al. (2022)
Aptamer	Fluorescence analysis	5 × 10^4^–1×10^6^	37	Fetal bovine serum	Huang et al. (2016)
Aptamer	Colorimetry	0.05–0.8	0.0131	Serum	Kongpreecha et al. (2023)
Aptamer	PEC	0.001–100	1.8 × 10^−4^	Serum	Wu et al. (2023)
Aptamer	PEC	1–500	0.12	Serum	Yao et al. (2020), p.17

## 4 Conclusion and future perspectives

E2 plays a vital role in cell growth, development and differentiation, reproductive system development, bone integrity maintenance, vascular system protection, and central nervous system regulation. Biosensors for detecting E2 have been in development for many years. The earliest sensitive detection of E2 was based on enzyme-linked immunity. However, when immobilizing the enzyme, embedding of the active site frequently occurs, thus affecting the results. And the enzymes that are easily affected by the PH, temperature and other factors in the detection environment are gradually unable to meet the continuous improvement of clinical and scientific research requirements. With the rapid development of antibody library screening techniques, recombinant antibody techniques and other technologies, antibodies with high specificity and affinity can be obtained to detect targets, and can mitigate the risk of inactivation or shedding caused by complex detection environments to a certain extent. Moreover, the antibody-based E2 sensor has a variety of energy-changing interfaces for antibody attachment and multiple detection mechanism to choose. Further in order to realize the real-time monitoring of E2, the appearance of aptamer, to some extent, overcomes the problems of temperature and pH sensitivity reduction, biological activity reduction, limited shelf life, an irreversible degeneration of other biometric molecules mentioned above, and complete E2 detection with various transducers.

Furthermore, the extensive application of new functional nanomaterials in various biosensors is mentioned during the investigation of this paper. Whether modified bioreceptors or energy exchange interfaces, functional nanomaterials improve the sensing performance from signal conversion rate, signal amplification, and biocompatibility. The application of nanomaterials in E2 biosensor mainly include two ways. The first is to modify the bioreceptor for immobilization or as a conversion medium for energy form to convert the change of E2 concentration into a detectable signal. The second is to modify the surface of the transducer for fixing the bioreceptor or to improve the signal quality. The applications of various nanomaterials in electrical and optical biosensors are the most abundant. Some of the most commonly used nanomaterials are metals, carbon-based materials, conductive polymers (CPs), and conducting composites (Li et al., 2016). These conducting materials have been prepared into nano-scale materials such as nanoparticles, nanorods, nanotubes, nanofibers, and nanowires. Metal nanoparticles possess better porous surface areas, and excellent conductivity, including AuNPs, silver nanoparticles, copper nanoparticles. Moreover, due to the nano-size effect, metal nanomaterials with different shapes and sizes can improve different sensing effects. Carbon-based materials, encompassing graphenes, carbon nanotubes (CNTs) and others, have the potential to enhance the functioning of biosensors, attributed to their broad potential window, outstanding electrochemical stability, substantial mechanical resilience, and compatibility with biological systems (Haroon and Stine, 2023). CPs are easy to synthesize and have good biocompatibility. In addition, their conjugated skeleton can be used to form a network to improve the electron transport characteristics. At present, the commonly used CPs include polypyrrole (PPy), polyurethane, polyaniline (PANI). In addition, different types of nanomaterials can be used in combination to provide a larger specific surface area, functional groups, mechanical properties and good biocompatibility for biosensors.

Even though biosensors for E2 detection are well developed, no commercially established product exists for E2 biosensors, and overcoming the constraints of detection scenarios remains uneasy. The following development points are proposed, hoping to help the further development of E2 biosensors.

(1) Employ various biometric molecules to enhance specificity. In addition to selecting biometric molecules from the perspective of specificity and fixation mode, they can also be cross-selected to detect E2. For already commercial platforms, one key feature of most is the use of a “sandwich-type biosensor” platform, which allows for very stable and sensitive signals to be generated, and can be coupled with other protocols to amplify the signal (Kim et al., 2023). For example, the combination of enzyme and antibody, aptamer and antibody, further increases the specificity of the E2 sensor.
(2) Integrate sampling and diagnosing to continuously dynamic monitor *in vivo* or *in vitro*. At present, detecting E2 requires multiple separation processes, such as sampling and sample pretreatment. Additionally, the test results depend on the standardized method of determination and the sample conditions, including but not limited to the extraction method, storage time, and sample environment. Therefore, developing an integrated testing platform which can continuously dynamic monitoring E2 is necessary to make testing more convenient. For *in vitro* diagnostics, paper chips are one of the most commonly used integrated biosensor devices, including LFAs, microfluidic/electrochemical paper-based analytical devices (μPADs/ePADs). These biosensors are not restricted by location. However, reproducibility of these devices remains hurdles due to the nature of paper and its fibers (Kim et al., 2023). Furthermore, for E2-related diseases, *in vivo*, real-time monitoring is needed in practical clinical applications. But there is still no portable biosensor to monitor E2 in real time. Detecting E2 *in vivo* requires 1) Selective suppression of false signals caused by interference existing in the complex environment *in vivo*; 2) No reagent operation, except the reagents provided by the organism *in situ*, without any exogenous reagents; 3) Reversible reaction to achieve dynamic measurement. There currently needs to be a general method for integrating this recognition into sensors that support real-time *in vivo* detection of E2. According to the various E2 biosensors introduced above, it is one of the effective methods to select aptamers as bioreceptor and electrode as transducer to realize real-time monitoring of E2 concentration. (3) Integrate multiple functions to extend application scenarios. We can add other functions to the biosensor depending on the practical application of the E2 sensor in the environment and disease. For example, in addition to the function of concentration detection, the degradation function can also be added to E2 detection in the environment (Xiong et al., 2020), and the drug delivery function can be added to disease detection (Dauphin-Ducharme et al., 2019). If commercialized products are further advanced, perhaps combining smartphones with detection platforms to provide reliable data into artificial intelligence (AI) to obtain better signal results is also a good option (Kim et al., 2023).
